# Naldemedine-induced perforation of a diverticulum in the sigmoid colon of a patient with opioid-related constipation: a case report

**DOI:** 10.1186/s40780-024-00371-9

**Published:** 2024-08-15

**Authors:** Hayato Yokota, Yumiko Akamine, Mizuki Kobayashi, Takuro Kitabayashi, Misato Horie, Tentaro Endo, Takechiyo Yamada, Masafumi Kikuchi

**Affiliations:** 1 Department of Pharmacy, Akita University Hospital, 1-1-1 Hondo, Akita, 010-8543 Japan; 2 Department of Otorhinolaryngology & Head and Neck Surgery, Akita University Graduate School of Medicine, Akita, Japan; 3 Department of Gastroenterological Surgery, Akita University Graduate School of Medicine, Akita, Japan

**Keywords:** Constipation, Diverticulum, Intestinal perforation, Naldemedine, Peripherally acting μ-opioid receptor antagonists

## Abstract

**Background:**

Naldemedine is an orally available peripherally acting μ-opioid receptor antagonist approved to treat opioid-induced constipation (OIC). It is contraindicated for patients with known or suspected gastrointestinal obstruction to protect against naldemedine-induced perforation. Here, we report a clinical case of suspected perforation of a diverticulum in the sigmoid colon associated with naldemedine.

**Case presentation:**

The patient was a 65-year-old man with a history of oral cancer who had been prescribed oxycodone (20 mg/day) for cancer pain. On day 0, the patient started naldemedine 0.2 mg once daily before bedtime for OIC. The dose of oxycodone was increased for pain control up to 60 mg/day. On day 35 of naldemedine treatment, the patient developed fever and abdominal pain, and his frequency of defecation had decreased. Initial laboratory results showed a C-reactive protein (CRP) level of 28.5 mg/dL and white blood cell (WBC) count of 13,500/µL. On day 37, the patient still had tenderness in his lower abdomen. Abdominal computed tomography revealed free air in the abdominal cavity suggesting an intestinal perforation. A Hartmann procedure was performed. Histopathological findings showed numerous diverticula in the sigmoid colon, some of which were perforated.

**Conclusions:**

These results suggest that the effects of OIC may have compressed the intestinal tract, which was followed by naldemedine-activation of peristalsis, which led to the onset of intestinal perforation. In patients with pre-existing diverticular disease, we should monitor for increased WBC counts and CRP levels after the initiation of treatment with naldemedine, and consider performing appropriate tests early in the event of abdominal complaints.

## Introduction

Chronic constipation often negatively impacts patients’ quality of life and can lead to physical disability. Opioid-induced constipation (OIC) is the most common side effect of opioids, which are widely used for pain in cancer and non-cancer patients and can cause chronic constipation. A reported 60%─90% of cancer patients taking opioids have chronic constipation [1]. Opioids bind to opioid receptors in the central nervous system to provide analgesia, and bind to μ-opioid receptors in the gastrointestinal tract. The latter activity has been reported to cause decreased intestinal peristalsis and decreased secretion of intestinal fluids and increased absorption of water, which results in constipation [2].

Naldemedine has a highly polar side chain, which reduces its penetration of the brain-blood barrier. It is a selective antagonist of peripheral μ-opioid receptors, making it an effective therapeutic agent for OIC [3]. The most frequent side effects of naldemedine are gastrointestinal disturbances such as abdominal pain, diarrhea, and nausea during the early stages of administration [4]. Other side effects are not well known. Methylnaltrexone bromide, a peripheral opioid receptor antagonist, has been reported to cause fatal gastrointestinal perforation after administration [5]. Therefore, and according to the package insert, naldemedine, a drug with a mechanism of action similar to that of methylnaltrexone bromide, is contraindicated in patients with or suspected of having a gastrointestinal obstruction [6]. Moreover, the package insert lists the potential risks of gastrointestinal perforation, which include localized or diffuse reduction in the structural integrity of the walls of the gastrointestinal tract (e.g., peptic ulcer disease and diverticular disease). To our best knowledge, however, there have been no reports from domestic or international clinical trials on gastrointestinal perforations associated with naldemedine. Here, we report a patient with OIC who developed perforations in his sigmoid colon during treatment with naldemedine.

## Case presentation

The patient was a 65-year-old man with stage IV (T4N1M0) cancer in the floor of his mouth and a history of bipolar disorder and Parkinson disease. The patient was an ex-smoker who had smoked up to 20 cigarettes per day for 40 years, but had stopped smoking 5 years before his current admission for oral cancer. He was admitted to the hospital for chemoradiotherapy for his oral cancer. The drugs used on admission were tramadol (150 mg/day) and naproxen (300 mg/day) for cancer pain, esomeprazole (20 mg/day) for decreasing the secretion of gastric acid, lamotrigine (50 mg/day) and clonazepam (1 mg/day) for bipolar disorder, and levodopa/carbidopa (200 mg/day) for Parkinson disease. On admission, the patient was changed from tramadol (150 mg/day) to extended-release oxycodone (20 mg/day) because of worsening cancer pain. Owing to the decreased frequency of defecation, the patient was started on sennoside (24 mg/dose) and bisacodyl (10 mg/dose) before bedtime, as needed. The patient was then started on naldemedine (0.2 mg, day 0) once daily along with his laxatives for OIC before bedtime.

The patient underwent chemoradiotherapy. The standard-of-care cisplatin regimen, which consisted of 3 weekly courses of high-dose cisplatin at 100 mg/m^2^ per dose, was adjusted in accordance with the patient’s renal function. He received 80 mg cisplatin per dose intravenously and a total radiation dose of 70 Gy. The patient received antiemetic therapy consisting of a combination of palonosetron (0.75 mg), fosnetupitant (235 mg), and dexamethasone (9.9 mg) starting from the initiation of chemotherapy. Because of mucositis induced by chemoradiotherapy, the oxycodone dose was gradually increased after the start of treatment to a maximum of 60 mg/day on day 28. On day 31, the patient underwent his second course of the cisplatin regimen.

**Fig. 1:**
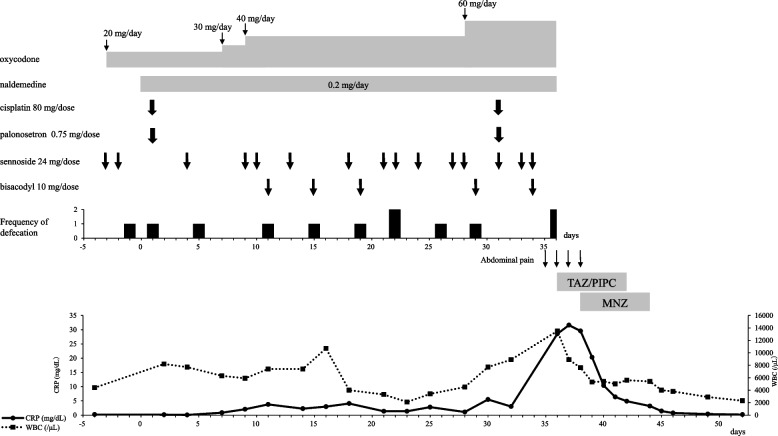
Clinical course of the patient. CRP, C-reactive protein; MNZ, metronidazole; TAZ/PIPC, tazobactam/piperacillin; WBC, white blood cell

On day 35, the patient had a fever of 38.5 °C and abdominal pains, and became increasingly bedridden. His frequency of defecation decreased from 2 to 3 times to less than once a week. The patient exhibited grade 2 constipation according to the Common Terminology Criteria for Adverse Events, version 5.0., with a Bristol Stool Scale score of 2 to 3. Laboratory testing revealed an elevated peripheral blood white blood cell (WBC) count (13,500 cells/μL) and a C-reactive protein (CRP) level of 28.5 mg/dL (Fig. 1). The patient was started on empirical antimicrobial therapy consisting of intravenous tazobactam/piperacillin (TAZ/PIPC, 13.5 g/day). Twenty-four hours later he complained of abdominal distention and tenderness of the entire lower abdomen. Abdominal computed tomography (CT) revealed multiple diverticula in the sigmoid colon, free air in the abdominal cavity, and fluid retention around the sigmoid colon, which was assumed to be the leaked contents of the intestinal tract. Emergent surgery was performed for acute peritonitis suspected to have been caused by perforation of a sigmoid colon diverticulum.

**Fig. 2:**
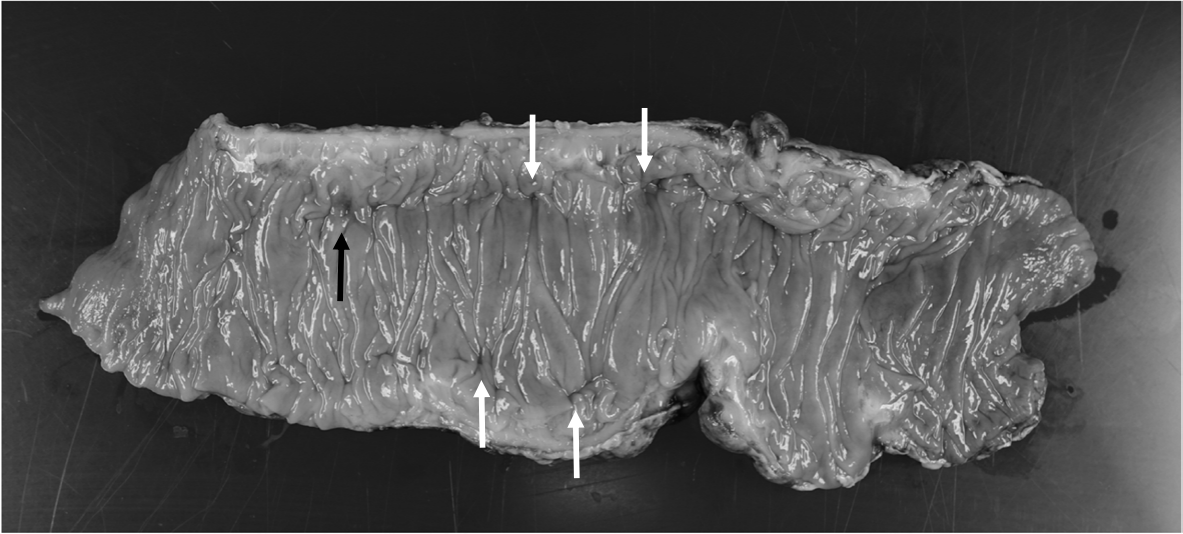
Resected specimen. The surgical specimen shows a diverticular perforation (black arrow) and multiple diverticula (white arrows)

A perforation was found on the anterior wall of the sigmoid colon, which was washed and drained. A Hartmann colostomy was performed. Histopathological findings revealed numerous diverticula, some of which showed diverticulitis with perforations (Fig. 2). Fibrin precipitation, inflammatory cell infiltration, and congestion were observed on the serosal surface. The findings were diagnosed as peritonitis with perforations associated with diverticulitis. The patient’s pain control was changed from oral oxycodone to fentanyl injections, and other drugs, including naldemedine, were discontinued. Based on the results of a culture of ascitic fluid, the patient received metronidazole (1.5 g/day for 7 days) in addition to his treatment with TAZ/PIPC.

The patient’s fever, CRP levels, and WBC counts gradually resolved after surgery. The drain tubes in the left and right subdiaphragmatic area, Douglas pouch, and around the sigmoid colon were removed. His medications were resumed, but the patient was administered extended-release morphine 40 (mg/day) via nasogastric tube for pain, instead of fentanyl by injection. The hospital pharmacist suggested that naldemedine should not be restarted because it could have been the cause of the intestinal perforation. Therefore, the patient was started on oral magnesium oxide at 330 mg three times daily to treat his OIC, and sennoside was restarted at 24 mg once daily as needed. The patient’s postoperative course was uneventful, and he was transferred to the local hospital for palliative care on day 73.

## Discussion

Mortality due to intestinal perforation is one of the most serious side effects of a peripherally acting μ-opioid receptor antagonist such as naldemedine. It has been reported to occur in 16.9%─33.3% of patients [7, 8]. Therefore, naldemedine is contraindicated in patients with known or suspected gastrointestinal obstruction or at increased risk for recurrent obstruction because of the risk of gastrointestinal perforation [6]. Our patient did not have a history of gastrointestinal obstruction, but diverticulitis was found at surgery, suggesting that the gastrointestinal tract was vulnerable. Therefore, we suggest that the administration of naldemedine led to intestinal perforation, as a result of the relative activation of intestinal function by the antagonistic effect of naldemedine on the μ-opioid receptors in the intestinal tract.

Opioids have been reported to act directly on the μ-opioid receptors of intestinal neurons to induce constipation by suppressing peristalsis through contraction of the circular muscle layer [9]. Since contractions are stronger in the distal regions (rectum, distal and transverse colon) than proximal regions (proximal colon, jejunum, and ileum), naldemedine may have antagonized the contractions in those regions. The intestinal perforation in our patient occurred in the distal colon near the rectum, suggesting that the release of contractions by the circular muscle layer caused by naldemedine led to a relative activation of peristalsis, followed by the onset of intestinal perforation. Moreover, peripherally restricted opioid receptor antagonists may manifest prokinetic activity [10, 11]. Endogenous opioids mediate inhibition of peristalsis in the guinea pig intestine via μ- and κ-opioid receptors [12]. Naldemedine has a potent antagonist activity against μ- and κ-opioid receptors [13]. Therefore, naldemedine may have inhibited the suppression of opioid-induced intestinal motility and promoted gastrointestinal motility. Meanwhile, stimulant laxatives, including sennoside and bisacodyl, act locally at the nerve plexus of smooth muscle in the intestine to promote colonic motility. Long-term or high-dose stimulant laxatives have been shown to cause morphological changes, neuronal damage, and functional impairment of the intestine [14]. However, in our case, they were administered over a short-term period and used at recommended therapeutic doses. Moreover, the chronic consumption of anthraquinone laxatives containing senna could have led to melanosis coli [15, 16], which was not confirmed by the histopathological or surgical findings.

To the best of our knowledge, this is the first report describing an intestinal perforation induced by the administration of naldemedine. There have been reports of gastrointestinal perforation following administration of methylnaltrexone bromide, which is an analog of naldemedine [5, 17]. Similarly, there have been case reports of stercoral perforation associated with the administration of the combination of buprenorphine and naloxone, which is an opioid antagonist [18]. The perforations described in these reports occurred immediately after or less than 1 week after the start of the administration of the drug, which is different from the timing of perforation in our patient. The rate of dissociation of the binding of naldemedine to the µ-opioid receptors that is caused by noncompetitive antagonism has been reported to be slower than that of *N*-methylnaltrexone or naloxone [13, 19]. The differences between the rates of dissociation have led to differences between the times of onset of peripheral withdrawal symptoms such as diarrhea from various μ-opioid receptor antagonists. Therefore, the time of the onset of side effects in our patient may also have been delayed in relation to the intestinal perforation that occurred after naldemedine administration.

Naldemedine was administered to our patient before bedtime. Administration of naldemedine without food has been reported to cause a 1.5-fold increase in maximal plasma concentration compared to the administration of naldemedine with food [6]. The concentrations of naldemedine in the intestinal tract may have been temporarily high, which may have contributed to the greatly enhanced peristaltic movements. However, the extent to which the timing of the administration of naldemedine in relation to the timing of food intake would be affected in clinical practice remains unknown.

The histopathological findings of our patient revealed a large number of diverticula. Patients with diverticulitis have been reported to develop complications, including perforation, stricture, fistula, and abscesses [20]. In addition, smoking is also a risk factor for severe colonic diverticulitis with perforations [21, 22], and our patient had a long history of smoking. Therefore, he was considered to be at risk for diverticulitis with perforations. In other words, the risk of intestinal perforation during administration of naldemedine should be considered in patients with pre-existing diverticular disease.

The patient had a decreased frequency of defecation one week before the onset of abdominal pain. Constipation has been reported to be another risk factor for perforation [23]. Our patient had OIC, Parkinson syndrome, and received palonosetron, a 5-HT_3_ receptor antagonist used during chemotherapy, which may have contributed to his chronic constipation [24, 25].

In clinical practice, the presence of diverticular disease in a patient may be unknown before the administration of naldemedine. Moreover, the incidence of diverticulitis increases with age [26]. Therefore, clinicians should consider changing to alternative medications rather than continuing the prolonged use of ineffective laxatives especially in elderly patients. Moreover, clinicians should minimize the use of medications that may induce constipation. In addition to peripheral μ-opioid receptor antagonists, other effective drugs for OIC in Japan include osmotic laxatives, stimulant laxatives, and lubiprostone, according to the Evidence-Based Clinical Practice Guidelines for Chronic Constipation 2023 [27]. The patient had a decreased frequency of defecation one week before the onset of abdominal pain. When constipation persists and does not improve despite naldemedine administration, a change to another laxative should be considered. In this case, magnesium oxide was started after the discontinuation of naldemedine, which improved the frequency of defecation. Furthermore, for constipated patients taking opioids for pain, a change to fentanyl, an opioid that has been reported to have less impact on constipation [28], should also be considered.

A rapid increase in the CRP levels and WBC counts was seen in our patient after the onset of abdominal pain, which was shown on abdominal CT to be an intestinal perforation. Because elevated markers of inflammation such as CRP and WBC have been reported to be predictive of perforation in patients with acute sigmoid diverticulitis [29], it is important to monitor for rapid increases in CRP levels and WBC counts during treatment with naldemedine.

This case report has limitations. The patient had both diverticulitis and constipation, which may have influenced the development of intestinal perforation. Smoking, a risk factor for diverticulitis, has been reported to be a risk factor for oral cavity and pharyngeal cancer [30]. Further research is needed to investigate the potential association between oral cancer and naldemedine-induced intestinal perforation. Additionally, because naldemedine is used to treat OIC, it was difficult to completely exclude the effects of constipation on perforation. Nevertheless, naldemedine is likely to be commonly prescribed in clinical practice for patients with OIC. Therefore, we think that this case report contains clinically significant findings.

In conclusion, we reported a patient with a diverticular perforation in his sigmoid colon that occurred after naldemedine administration. When using naldemedine for OIC in patients with pre-existing diverticular disease, we should be aware of the risk for intestinal perforation. During naldemedine administration, we should evaluate for abdominal tenderness and carefully monitor for increasing WBC counts and CRP levels. Furthermore, during the treatment of OIC, the selection of an appropriate laxative or change to another opioid should be considered frequently, depending on the patient's condition.

## Data Availability

Data sharing is not applicable to this article as no datasets were generated or analyzed during the current study.

## Abbreviations

CT: Computed tomography
CRP: C-reactive protein
OIC: Opioid-induced constipation
TAZ/PIPC: Tazobactam/piperacillin
WBC: White blood cell
